# Low humoral and cellular immune responses early after breakthrough infection may contribute to severe COVID-19

**DOI:** 10.3389/fimmu.2023.1106664

**Published:** 2023-03-22

**Authors:** Chan Mi Lee, Pyoeng Gyun Choe, Chang Kyung Kang, Eunyoung Lee, Kyoung-Ho Song, Ji Hwan Bang, Eu Suk Kim, Hong Bin Kim, Nam Joong Kim, Hang-Rae Kim, Youngju Kim, Chang-Han Lee, Hyun Mu Shin, Sang-Won Park, Wan Beom Park, Myoung-don Oh

**Affiliations:** ^1^Department of Internal Medicine, Seoul National University College of Medicine, Seoul, Republic of Korea; ^2^Department of Internal Medicine, Seoul National University Hospital, Seoul, Republic of Korea; ^3^Department of Internal Medicine, Seoul National University Boramae Medical Center, Seoul, Republic of Korea; ^4^Department of Internal Medicine, Seoul National University Bundang Hospital, Seongnam, Republic of Korea; ^5^Department of Anatomy & Cell Biology and Biomedical Sciences, Seoul National University College of Medicine, Seoul, Republic of Korea; ^6^BK21 FOUR Biomedical Science Project, Seoul National University College of Medicine, Seoul, Republic of Korea; ^7^Department of Biomedical Sciences, Seoul National University College of Medicine, Seoul, Republic of Korea; ^8^Department of Pharmacology, Seoul National University College of Medicine, Seoul, Republic of Korea; ^9^Wide River Institute of Immunology, Seoul National University, Hongcheon, Republic of Korea

**Keywords:** SARS-CoV-2, breakthrough infection, COVID-19, immune response, antibody

## Abstract

**Background:**

Little is known about the immune determinants for severe coronavirus disease 2019 (COVID-19) in individuals vaccinated against severe acute respiratory syndrome coronavirus 2. We therefore attempted to identify differences in humoral and cellular immune responses between patients with non-severe and severe breakthrough COVID-19.

**Methods:**

We prospectively enrolled hospitalized patients with breakthrough COVID-19 (severe and non-severe groups) and uninfected individuals who were vaccinated at a similar time (control group). Severe cases were defined as those who required oxygen therapy while hospitalized. Enzyme-linked immunosorbent assays and flow cytometry were used to evaluate humoral and cellular immune responses, respectively.

**Results:**

Anti-S1 IgG titers were significantly lower in the severe group than in the non-severe group within 1 week of symptom onset and higher in the non-severe group than in the control group. Compared with the control group, the cellular immune response tended to be diminished in breakthrough cases, particularly in the severe group. In multivariate analysis, advanced age and low anti-S1 IgG titer were associated with severe breakthrough COVID-19.

**Conclusions:**

Severe breakthrough COVID-19 might be attributed by low humoral and cellular immune responses early after infection. In the vaccinated population, delayed humoral and cellular immune responses may contribute to severe breakthrough COVID-19.

## Introduction

Since the coronavirus disease 2019 (COVID-19) pandemic was declared, various types of vaccines against severe acute respiratory syndrome–coronavirus 2 (SARS-CoV-2) have been rapidly developed. Vaccination has been the primary strategy for containing the COVID-19 pandemic, and therefore, several COVID-19 vaccines have been rapidly rolled out.

Based on real-world data together with clinical trials, vaccination was associated with reduced risks of COVID-19–related hospital admission and mortality (1–3) as well as reduced risks of symptomatic and severe COVID-19 (4, 5). However, despite the high vaccine efficacy and effectiveness against COVID-19 (6–9), breakthrough COVID-19 cases have continued to emerge (10). While the majority of breakthrough COVID-19 have been mild or moderate (11), severe or fatal cases have not been rare (12–14). In addition, breakthrough COVID-19 cases have also been reported even after booster doses (15).

However, little is known about the immune response to breakthrough infection. Bergwerk and colleagues reported a correlation between peri-infection antibody titers and breakthrough COVID-19 (10), but a subsequent study found no difference in post-vaccination neutralizing antibody titers between controls and patients with breakthrough infection (16). Another previous study reported that the antibody titers declined after a second vaccination but were sharply elevated in breakthrough COVID-19 cases (17). These previous studies were limited, however, by their focus on healthy healthcare personnel and their analysis of only mild breakthrough COVID-19 cases.

It has become crucial to characterize the immune response in cases of breakthrough COVID-19, especially severe cases, because the vaccinated population has become the mainstay, and the incidence and medical burden of breakthrough COVID-19 continue to persist. This study therefore attempted to identify differences in humoral and cellular immune responses according to severity among hospitalized patients with breakthrough COVID-19.

## Materials and methods

### Study population and design

From May 2021 to January 2022, we prospectively enrolled study participants aged ≥18 years with reverse transcription–polymerase chain reaction (RT-PCR)–confirmed SARS-CoV-2 infection who were admitted to the biocontainment units of Seoul National University Hospital or Boramae Medical Center. Uninfected vaccinated individuals were also enrolled. The uninfected vaccinated individuals were enrolled from the non-hospitalized general population. Breakthrough COVID-19 was defined by the presence of COVID-19 symptoms and RT-PCR–confirmed diagnosis of COVID-19 more than 14 days after at least one vaccine dose. Patients with breakthrough COVID-19 were divided into a severe group who required oxygen therapy and a non-severe group who did not require supplemental oxygen therapy during hospitalization (18). The uninfected vaccinated individuals served as a control group.

Within 1 week of onset of symptoms, blood samples were collected from patients hospitalized with COVID-19. Additional serial samples were obtained from a portion of the patients. Blood samples were also collected from unvaccinated patients with severe COVID-19 and uninfected vaccine recipients. Data were collected regarding demographic characteristics, vaccination type and status, days from vaccination to symptom onset, Charlson’s comorbidity index, underlying diseases, and clinical outcomes. Fully vaccinated patients were defined as those with COVID-19 diagnosis more than 14 days after completion of the recommended vaccination regimen.

### Measurement of anti-S1 immunoglobulin G by enzyme-linked immunosorbent assay

Anti-S1 (spike subunit) IgG titer was semi-quantitatively measured using an enzyme immunoassay kit (Euroimmun, Lübeck, Germany) according to the manufacturer’s protocol. Optical density (O.D.) ratios were interpreted as follows according to the instructions: <0.8, negative; ≥0.8 to <1.1, borderline; and ≥1.1, positive.

### Measurement of anti–receptor-binding domain IgG by ELISA

The binding activity of serum antibodies to SARS-CoV-2 receptor-binding domain (RBD) proteins was determined using an ELISA (19, 20). ELISA plates were coated with 100 ng/well of RBD protein, blocked with phosphate-buffered saline (PBS) supplemented with 3% bovine serum albumin, and incubated with diluted serum (1:200) for 2 h. Bound antibodies were detected using horseradish peroxidase–conjugated goat anti-human IgG (Fc) (#ARG23874, 1:12,000, Arigo Biolaboratories, Hsinchu, Taiwan). After washing with PBST three times, 50 μL of 3,3’,5,5’-tetramethyl benzidine was added, followed by the addition of 50 μL of 2 M H_2_SO_4_ to stop the reaction. Absorbance was measured at 450 nm using an Infinite M200 PRO (TECAN, Zurich, Switzerland).

### Collection of peripheral blood mononuclear cells, antigen stimulation, and flow cytometry

After whole blood was drawn into heparin vacutainers (Becton Dickinson, NJ, USA), PBMCs were purified using Ficoll-Hypaque (GE Healthcare Life Sciences, Piscataway, NJ, USA). PBMCs were stored in serum-free cryopreservation medium (Cellbanker 2; Zenoaq, Japan) in liquid nitrogen containers until further use.

After thawing, cells were cultured in the presence of 1% penicillin/streptomycin (Thermo Fisher Scientific, Waltham, MA, USA) in complete RPMI-1640 medium containing 10% fetal bovine serum. Thereafter, 1 × 10^6^ PBMCs/mL were stimulated with 0.06 nmol/mL PepTivator^®^ SARS-CoV-2 Prot_S Complete (*i.e*., whole-spike Ag), PepTivator^®^ SARS-CoV-2 Prot_S B.1.617.2 wild-type (WT) reference (*i.e*., WT Ag), or PepTivator^®^ SARS-CoV-2 Prot_S B.1.617.2 Mutation Pool (*i.e*., Delta Ag) (Miltenyi Biotec, Bergisch Gladbach, Germany) for 24 h. Medium alone was used as a negative control. In addition to the antigens, Brilliant Blue 515–anti-human CD4 (clone RPA-T4) antibodies (BD Biosciences, San Jose, CA, USA) for staining CD4 and anti-human CD28/CD49d (clone L293/L25, BD Biosciences) antibodies for co-stimulation were simultaneously added. For the final 4 h of antigen stimulation, cells were treated with BD GolgiPlug^®^ (brefeldin A) and BD GolgiStop^®^ (monensin) (all from BD Biosciences).

Dead cells were stained with LIVE/DEAD (Thermo Fisher Scientific) after stimulation. Cells were permeabilized before incubation with peridinin chlorophyll protein complex–anti-human CD8 (clone SK1), Brilliant Violet (BV) 510–anti-human CD3 (clone CHT1), BV605–anti-human CD69 (clone FN50), BUV395–anti-human CD137 (clone 4B4-1), phycoerythrin–indotricarbocyanine (Cy7)–anti-human IFN-γ (clone B27), allophycocyanin–anti-human interleukin(IL)-2 (clone 5344.111), phycoerythrin–anti-human tumor necrosis factor-α (clone Mab11), and BV421–anti-human IL-4 (clone MP4-25D2) antibodies (all from BD Biosciences). Each sample was treated with BD Horizon Brilliant Stain Buffer (BD Biosciences). In each experiment, compensation beads (UltraComp eBeads, Thermo Fisher Scientific) and unstimulated cells were used for compensation. Flow cytometric data were acquired on a FACSymphony system (BD Biosciences) and analyzed using FlowJo software (version 10.7.1; TreeStar, Ashland, OR, USA).

The frequencies of SARS-CoV-2–specific activation-induced marker^+^ (AIM^+^, CD69^+^CD137^+^) CD4^+^ T cells or CD8^+^ T cells and cytokine-producing CD137^+^CD4^+^ or CD137^+^CD8^+^ T cells were assessed (21). To analyze only the SARS-CoV-2–specific response, the percentages of target populations in specimens without antigen stimulation were subtracted from those in stimulated specimens (22). The flow cytometry gating strategy for SARS-CoV-2–reactive T cells and cytokine-producing T cells is shown in **Supplementary Figure 1**.

### Statistical analyses

The chi-squared test or Fisher’s exact test was used to compare categorical variables, and the Mann-Whitney *U* test was used to compare continuous variables. To identify risk factors for severe breakthrough COVID-19, variables with a *P* value of <0.10 in the univariate analysis were included in the multivariable logistic regression analysis. Statistical analyses were performed using SPSS Statistics, version 26.0 (IBM Corp., Armonk, NY, USA). *P* values <0.05 were considered statistically significant. Data are presented as median with interquartile range (IQR) and as dot plots. All graphs were generated using GraphPad Prism 9 (GraphPad Software, La Jolla, CA, USA).

## Results

### Study participants

A total of 108 breakthrough COVID-19 cases admitted within 1 week of the onset of symptoms were enrolled. The non-severe group included 79 (73.1%) patients, and the severe group included 29 (26.9%) patients. In addition, the control group included 22 uninfected individuals who completed the standard doses of SARS-CoV-2 vaccine (**Table 1**).

**Table1:** Baseline characteristics and clinical outcomes of patients with breakthrough COVID-19.

Characteristics	Control (*n* = 22)	Breakthrough COVID-19		*P* ^a^	*P* ^b^	*P* ^c^
		Non-severe (*n* = 79)	Severe (*n* = 29)			
Age, median (IQR), years	62 (47–73)	61 (47–68)	71 (63–78)	0.616	0.006	<0.001
Male, n (%)	19 (86.4)	41 (51.9)	19 (65.5)	0.004	0.091	0.207
BMI, median (IQR)*		24.1 (22.3–26.3)	24.0 (21.5–26.2)			0.886
Vaccination type, n (%)						
Adenoviral vector vaccines	10 (45.5)	39 (49.4)	14 (48.3)	0.745	0.842	0.920
mRNA vaccines	12 (54.5)	40 (50.6)	15 (51.7)**			
Vaccination status, n (%)						
Fully vaccinated	22 (100.0)	72 (91.1)	24 (82.8)	0.342	0.062	0.298
Partially vaccinated	0 (0.0)	7 (8.9)	5 (17.2)			
Days from vaccination to symptom onset, median (IQR)	55 (48–73)***	89 (54–120)	103 (58–157)	0.041	0.054	0.443
Days from symptom onset to first sampling, median (IQR)		4 (3–5)	5 (3–6)			0.072
Charlson’s comorbidity index, median (IQR)		2.0 (0.0–4.0)	4.0 (3.0–5.0)			<0.001
Underlying disease, n (%)						
Solid tumor		7 (8.9)	4 (13.8)			0.481
Hematologic malignancy		2 (2.5)	0 (0.0)			>0.999
Immunosuppressant use		9 (11.4)	6 (20.7)			0.223
Anti-S1 IgG in early phase, median (IQR), O.D. ratio	4.22 (2.46–6.13)	8.28 (5.17–10.25)	4.99 (1.38–9.02)	< 0.001	0.591	0.007
Clinical outcomes						
In-hospital mortality, n (%)		0 (0.0)	2 (6.9)			0.070
Admission duration, median (IQR), days		9 (7–10)	9 (8–12)			0.035

IQR, interquartile range; BMI, body mass index.

a P values between control and non-severe groups.

b P values between control and severe groups.

c P values between non-severe and severe groups.

* The BMI of the control group was not collected.

** One case was cross-vaccinated; the first vaccine was an adenoviral vector vaccine and the second vaccine was an mRNA vaccine.

*** Days from last vaccination to sampling.

The severe group was older (median [IQR], 71 [63–78] years vs. 61 [47–68] years, *P*<0.001) and had higher Charlson’s comorbidity index (median [IQR], 4.0 [3.0–5.0] vs. 2.0 [0.0–4.0], *P*<0.001) and longer duration of admission (median [IQR], 9 [8–12] vs. 9 [7–10], *P* = 0.035) than the non-severe group. Vaccination type and status did not significantly differ between the two groups. The control group was younger than the severe group (median [IQR], 62 [47–73] years vs. 71 [63–78] years, *P* = 0.006), and the interval between vaccination and sample collection was shorter in the control group than in the non-severe and severe groups.

### Humoral immune responses against SARS-CoV-2

**Figure 1:**
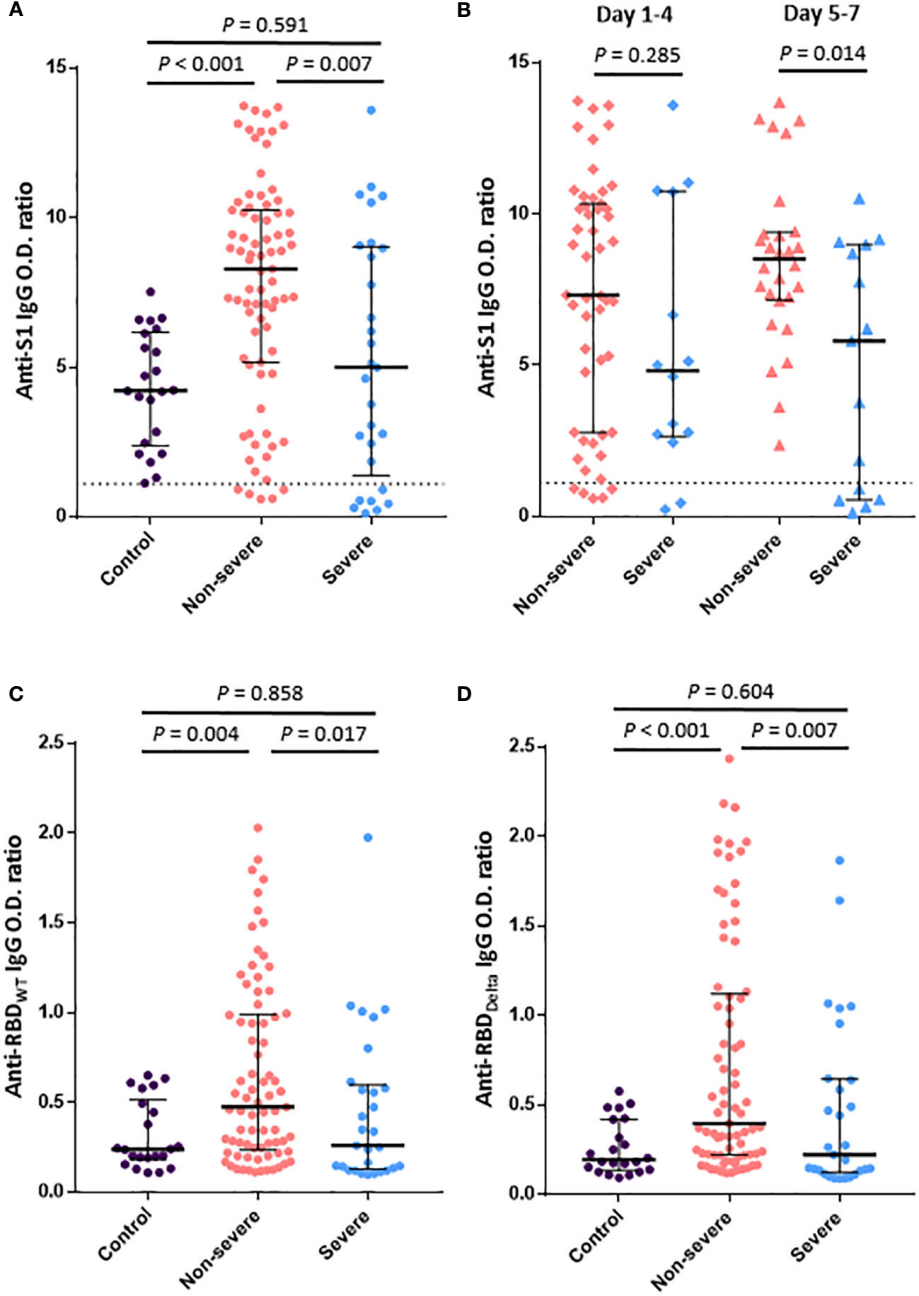
Humoral immune responses to SARS-CoV-2 within 1 week after symptom onset in patients with breakthrough COVID-19. **(A)**. Anti-S1 IgG antibody titers in the control (vaccinated subjects without infection), severe, and non-severe groups. **(B)**. Anti-S1 IgG antibody titers according to the number of days from symptom onset. **(C)**. IgG-binding activities to RBD_WT_. **(D)**. IgG-binding activities to RBD_Delta_. The dotted line shows the positive cutoff value of the anti-S1 IgG O.D. ratio. Vertical and horizontal lines indicate the median with the interquartile range.

Anti-S1 IgG titer within 1 week after symptom onset in breakthrough COVID-19 patients was significantly lower in the severe group than in the non-severe group (median O.D. ratio [IQR], 4.99 [1.38–9.02] vs. 8.28 [5.17–10.25], *P* = 0.007) (**Figure 1A**). Compared with the uninfected vaccinated control group, the anti-S1 IgG titer was significantly higher in the non-severe group (median [IQR], 4.22 [2.46–6.13] vs. 8.28 [5.17–10.25], *P*<0.001) but similar in the severe group (median [IQR], 4.22 [2.46–6.13] vs. 4.99 [1.38–9.02], *P* = 0.591). Anti-S1 IgG titer according to the number of days after symptom onset was significantly higher in the non-severe group than in the severe group on days 5–7 after symptom onset (median [IQR], 8.50 [7.15–9.39] vs. 5.80 [0.55–8.98], *P* = 0.014) but did not differ significantly between the two groups on days 1–4 after symptom onset (median [IQR], 7.31 [2.77–10.33] vs. 4.81 [2.64–10.73], *P* = 0.285) (**Figure 1B**).

Serum antibody responses to SARS-CoV-2 RBD_WT_ and RBD_Delta_ were compared between groups (**Figures 1C, D**), but two cases from the non-severe group were excluded due to shortage of samples. Titers of both anti-RBD_WT_ IgG and anti-RBD_Delta_ IgG were significantly lower in the severe group than in the non-severe group (median [IQR], 0.26 [0.13–0.60] vs. 0.48 [0.24–0.99], *P* = 0.017; 0.22 [0.13–0.64] vs. 0.40 [0.22–1.12], *P* = 0.007). Compared with the control group, the non-severe group showed significantly higher antibody responses to both RBD_WT_ and RBD_Delta_ (median [IQR], 0.24 [0.18–0.52] vs. 0.48 [0.24–0.99], *P* = 0.004; 0.19 [0.14–0.42] vs. 0.40 [0.22–1.12], *P*<0.001), but the severe group showed similar antibody responses to both RBD_WT_ and RBD_Delta_.

**Figure 2:**
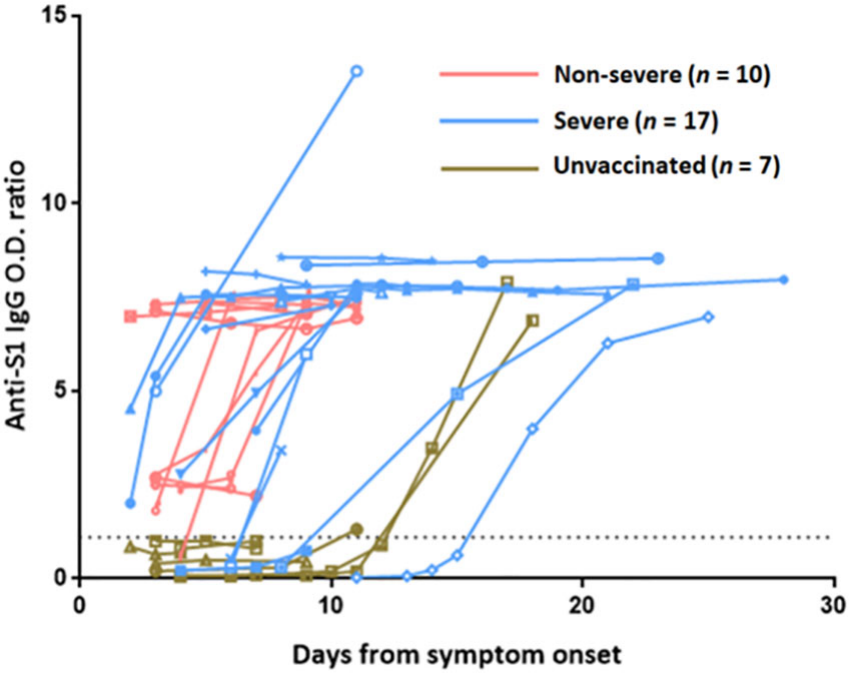
Anti-S1 IgG antibody kinetics over time after SARS-CoV-2 infection in vaccinated and unvaccinated patients. Serial antibody titers in vaccinated patients with non-severe (*n* = 10) and severe (*n* = 17) breakthrough COVID-19 and unvaccinated patients with SARS-CoV-2 infection (*n* = 7) are plotted. The dotted line shows the positive cutoff value of the anti-S1 IgG O.D. ratio.

The kinetics of antibody responses over time beginning at symptom onset were evaluated in 17 severe breakthrough cases, 10 non-severe breakthrough cases, and 7 unvaccinated severe cases (**Figure 2**). In non-severe breakthrough cases, the anti-S1 IgG titer was high at the time of symptom onset or elevated during the early phase of infection. However, the anti-S1 IgG titer increased later in some of severe breakthrough cases. In two severe breakthrough cases, the antibody titer began to increase 2 weeks after symptom onset, similar to unvaccinated severe cases. One of these two severe cases had received an immunosuppressant.

### Cell-mediated immune responses against SARS-CoV-2

T cell–mediated immune responses against the SARS-CoV-2 WT and Delta variant were assessed using flow cytometry in 10 non-severe breakthrough cases, 5 severe breakthrough cases, and 10 uninfected vaccinated individuals. Clinical data for the study participants whose cellular immune responses were analyzed are provided in **Supplementary Table 1**. Among severe cases, all PBMCs used in the analysis of cellular immune responses were isolated from blood samples collected before oxygen therapy.

**Figure 3:**
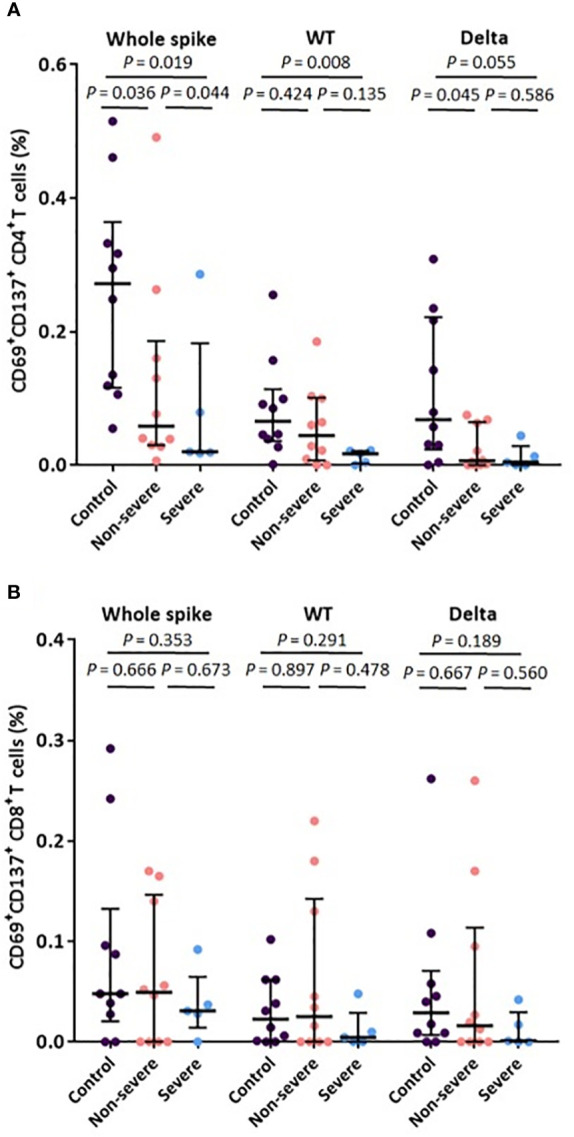
T cell responses within 1 week after diagnosis of breakthrough COVID-19. **(A)** AIM^+^ CD4^+^ T cells stimulated with the whole spike Ag, and the matched WT and Delta Ags. **(B)** AIM^+^ CD8^+^ T cells stimulated with the whole spike Ag, and the matched WT and Delta Ags. The control group denotes vaccinated subjects without breakthrough COVID-19. Vertical and horizontal lines indicate the median with the interquartile range.

Compared with uninfected vaccinated controls, the proportion of AIM^+^ CD4^+^ T cells specific for whole-spike Ag was significantly lower in both the non-severe breakthrough cases (median [IQR], 0.27% [0.12–0.36%] vs. 0.06% [0.03–0.19%], *P* = 0.036) and severe breakthrough cases (median [IQR], 0.27% [0.12–0.36%] vs. 0.02% [0.02–0.18%], *P* = 0.019) (**Figure 3A**). The proportion of AIM^+^ CD4^+^ T cells specific for the WT and Delta Ags tended to be lower in the severe group than in the non-severe group, although the difference was not statistically significant. In addition, the proportions of cytokine-producing CD4^+^ T cells tended to be lower in the severe group than in the non-severe group (**Supplementary Figure 2**). Although the differences were not statistically significant, the proportions of AIM^+^ CD8^+^ T cells specific for the WT and Delta Ags tended to be lower in the severe group than in the non-severe group (**Figure 3B**).

### Risk factors for severe breakthrough COVID-19

The univariate analysis identified older age, higher Charlson’s comorbidity index, and lower anti-S1 IgG titer in the early phase (≤1 week after symptom onset) as risk factors for severe breakthrough COVID-19 (**Table 2**). The multivariate analysis identified older age (adjusted odds ratio [aOR] 1.07, 95% confidence interval [CI] 1.01–1.12, *P* = 0.014) and low anti-S1 IgG titer in the early phase (aOR 0.88, 95% CI 0.77–0.99, *P* = 0.037) as independent risk factors for severe breakthrough COVID-19.

**Table 2:** Risk factors for severe breakthrough COVID-19.

Variable	Univariate		Multivariate	
	OR (95% CI)	*P*	aOR (95% CI)	*P*
Age	1.07 (1.03–1.12)	0.001	1.07 (1.01–1.12)	0.014
Sex				
Female	1.00			
Male	1.76 (0.73–4.26)	0.210		
BMI	1.03 (0.92–1.15)	0.603		
Vaccination type				
Adenoviral vector vaccine	1.00			
mRNA vaccine	1.05 (0.45–2.45)	0.920		
Vaccination status				
Partially vaccinated	1.00			
Fully vaccinated	0.47 (0.14–1.61)	0.227		
Days from vaccination to symptom onset	1.00 (0.99–1.01)	0.485		
Charlson’s comorbidity index	1.34 (1.11–1.63)	0.003	1.01 (0.77–1.34)	0.923
Underlying disease				
Solid tumor	1.65 (0.44–6.10)	0.456		
Hematologic malignancy	–	0.999		
Immunosuppressant use	2.03 (0.65–6.32)	0.222		
Anti-S1 IgG in early phase	0.85 (0.76–0.95)	0.006	0.88 (0.77–0.99)	0.037

OR, odds ratio; CI, confidence interval; aOR, adjusted odds ratio; BMI, body mass index.

The results of the multivariate linear regression model are shown in **Supplementary Table 2**. Among breakthrough COVID-19 patients, anti-S1 IgG titer was negatively correlated with severe COVID-19 (standardized β, −0.227, *P* = 0.025) and male sex (standardized β, −0.211, *P* = 0.029).

## Discussion

Compared with non-severe breakthrough COVID-19 patients, those with severe breakthrough COVID-19 exhibited significantly lower humoral immune responses and a trend toward lower cellular immune responses in the early phase of infection. In addition, we identified low anti-S1 IgG titer in the early phase as an independent risk factor for severe breakthrough COVID-19. Although previous studies identified immunologic factors associated with the occurrence of breakthrough COVID-19 (10, 23), our study analyzed immunologic factors that influence the severity of breakthrough infection.

We also investigated the relationship between various demographic characteristics and the severity of breakthrough infection and identified older age as an independent risk factor for severe breakthrough COVID-19. In a retrospective study, Suleyman and colleagues identified older age, together with underlying conditions such as cardiovascular disease and immunocompromised status, as a risk factor for hospitalization in breakthrough COVID-19 cases (24), which is consistent with our findings. Older age and multiple comorbidities are decisive risk factors for poor outcomes in unvaccinated patients infected with SARS-CoV-2 (25, 26). This suggests that certain critical factors associated with a poor outcome prior to vaccination may continue to be crucial predictors for severe breakthrough COVID-19.

The anti-S1 IgG titer differed between breakthrough COVID-19 patients and uninfected vaccinated controls. Although the interval between the last vaccination and sampling was shorter in the uninfected vaccinated control group than in the breakthrough infection groups (meaning that the control group exhibited less waning of antibodies over time), the anti-S1 IgG titer was higher in the breakthrough infection groups than in the uninfected vaccinated control group within 1 week after symptom onset. This finding suggests that memory B-cell responses to SARS-CoV-2 infection occurred within several days after symptom onset in patients with breakthrough infection. A previous study found that anti-S1 RBD IgG titer post-infection was significantly higher in mildly symptomatic patients than in those with no breakthrough infection or asymptomatic patients (27). This finding is also indicative of early immune boosting by symptomatic breakthrough infection.

The lower anti-S1 IgG antibody titer in the severe group compared with the non-severe group in the early phase after infection suggests that a poor memory B-cell response to SARS-CoV-2 infection might be related to progression of severe disease. Brosh-Nissimov and colleagues also reported that the anti-spike IgG titer was lower in fully vaccinated hospitalized COVID-19 patients with a poor outcome, but the difference was not statistically significant (28). In our study, the difference in anti-S1 IgG antibody titer between the non-severe and severe groups was prominent at 5–7 days after symptom onset. This suggests that a delay in antibody response of up to 1 week after symptom onset might be associated with severe COVID-19 after vaccination. A previous study found that peak viral load followed by a delayed increase in antibody response coincided with viral clearance after breakthrough infection, indicating a protective role of vaccination against severe COVID-19 (29). In addition, antibody kinetics assessed using serial samples revealed that memory B-cell responses were poor in the severe group; in two severe breakthrough cases, we did not observe any memory B-cell response until the antibody titer began to increase 2 weeks after symptom onset, similar to unvaccinated cases (30). These findings suggest that an adequate memory B-cell response in the early phase of breakthrough COVID-19 infection is critical to prevent progression to severe disease.

The cellular immune response is considered an important host factor affecting the severity of COVID-19. Hypofunction and a low number of T cells in the early phase have been associated with severe COVID-19 (31). In the present study, the severe group tended to exhibit a lower cellular immune response than the non-severe group, suggesting that a lower cellular immune response is associated with progression of severe breakthrough COVID-19.

Paniskaki and colleagues found that alpha variant–reactive CD4^+^ and CD8^+^ T-cell responses were very poor at disease onset in patients with moderate-to-critical breakthrough infection compared with uninfected vaccinated controls (32). Similarly, in the present study, cellular immune responses were lower in the breakthrough infection groups than in the uninfected vaccinated control group. This conflicts with the results of a previous study reporting that breakthrough cases exhibited a lower memory B-cell response but similar T-cell response compared with uninfected participants (33). These discrepancies might be due to differences in other host factors, as our study included hospitalized breakthrough COVID-19 cases with underlying medical conditions.

This study has several limitations. First, we could not obtain baseline samples before breakthrough COVID-19 to measure the immunologic response achieved purely by vaccination. Therefore, the immune responses after breakthrough COVID-19 in this study should be interpreted in consideration of contributions by both the vaccine-induced immune responses and immune boosting by natural infection. Second, the sample size for the investigation of cellular immune responses was relatively small, which precluded demonstration of statistical significance. Third, the sampling time was not consistent for each patient, even though patients in the early stage of infection were enrolled. Finally, the infecting virus was not analyzed; however, it is likely that most patients were infected with the Delta variant, considering that this variant was dominant during the study period.

In conclusion, we found that humoral and cellular immune responses in the early phase of infection were low in patients with severe breakthrough COVID-19 compared with non-severe patients. In the vaccinated population, delayed humoral and cellular immune responses, possibly due to poor memory B- or T-cell responses, may contribute to severe COVID-19.

## Data availability statement

The original contributions presented in the study are included in the article/**Supplementary Material**. Further inquiries can be directed to the corresponding authors.

## Ethics statement

The studies involving human participants were reviewed and approved by the Institutional Review Boards of Seoul National University Hospital (IRB no. 2104-182-1215) and Boramae Medical Center (IRB no. 20-2021-54). The patients/participants provided their written informed consent to participate in this study.

## Author contributions

HS, WP and S-WP conceptualized the study. CL, PG, and CK analyzed the data and drafted the initial manuscript. EL, K-HS, JB, EK, HK, NK, H-RK, YK, C-HL and M-DO made contributions to the manuscript. M-DO, WP and S-WP revised and edited the final manuscript. All authors contributed to the article and approved the submitted version.
