# Supplementary material for: Low humoral and cellular immune responses early after breakthrough infection may contribute to severe COVID-19
Source: Front Immunol. 2023 Mar 22;14:1106664. doi: 10.3389/fimmu.2023.1106664 (PMC10073433; doi:10.3389/fimmu.2023.1106664)
Supplement: Supplementary file 1 [file DataSheet_1.docx]

**Low humoral and cellular immune responses early after breakthrough infection may contribute to severe COVID-19**

**Supplement Contents**

Supplementary Table 1. -------------------------------------------------------------------- page 2

Supplementary Table 2. -------------------------------------------------------------------- page 3

Supplementary Figure 1. ------------------------------------------------------------------- page 4

Supplementary Figure 2A. ----------------------------------------------------------------- page 5

Supplementary Figure 2B. ----------------------------------------------------------------- page 6

**Supplement Figure Legends**

Supplementary Figure 1. Flow cytometry gating strategy for SARS-CoV-2 reactive T cells and cytokine-producing T cells.

Supplementary Figure 2. Cytokine producing T cell against SARS-CoV-2 within one week after diagnosis of breakthrough COVID-19. A. CD4^+^ T cells producing specific cytokines. B. CD8^+^ T cells producing specific cytokines. IFN-γ, interferon-γ; IL, interleukin; TNF-α, tumor necrosis factor-α. Vertical and horizontal lines indicate median with interquartile range.

Supplementary Table 1. Clinical information of study participants whose T cell responses were analyzed

| Characteristics | Control (*n* = 10) | Non-severe (*n* = 10) | Severe (*n* = 5) |
| --- | --- | --- | --- |
| Age, median (IQR), years | 70 (53-76) | 68 (61−76) | 64 (60−68) |
| Male, n (%) | 8 (80.0) | 8 (80.0) | 3 (60.0) |
| Vaccination type, n (%) |  |  |  |
| Adenoviral vector vaccines | 2 (20.0) | 6 (60.0) | 1 (20.0) |
| mRNA vaccines | 8 (80.0) | 4 (40.0) | 4 (80.0)* |
| Vaccination status, n (%) | | | |
| Fully vaccinated | 10 (100.0) | 9 (90.0) | 4 (80.0) |
| Partially vaccinated | 0 (0.0) | 1 (10.0) | 1 (20.0) |
| Days from vaccination to diagnosis, median (IQR) | 88 (54-140)** | 85 (46−93) | 103 (78−106) |

IQR, interquartile range;

* One case was cross-vaccinated; the first vaccine was an adenoviral vector vaccine and the second vaccine was an mRNA vaccine.

** Days from last vaccination to sampling.

Supplementary Table 2. Variables associated with anti-S1 IgG titers in multivariate linear regression model

| Variables | Total (*n* = 108) | |
| --- | --- | --- |
|  | Standardized β | *P* |
| Age | -0.063 | 0.553 |
| Sex, male | -0.211 | 0.029 |
| Vaccination type, mRNA vaccines | 0.009 | 0.928 |
| Days from vaccination to symptom onset | 0.029 | 0.765 |
| Severe COVID-19 | -0.227 | 0.025 |


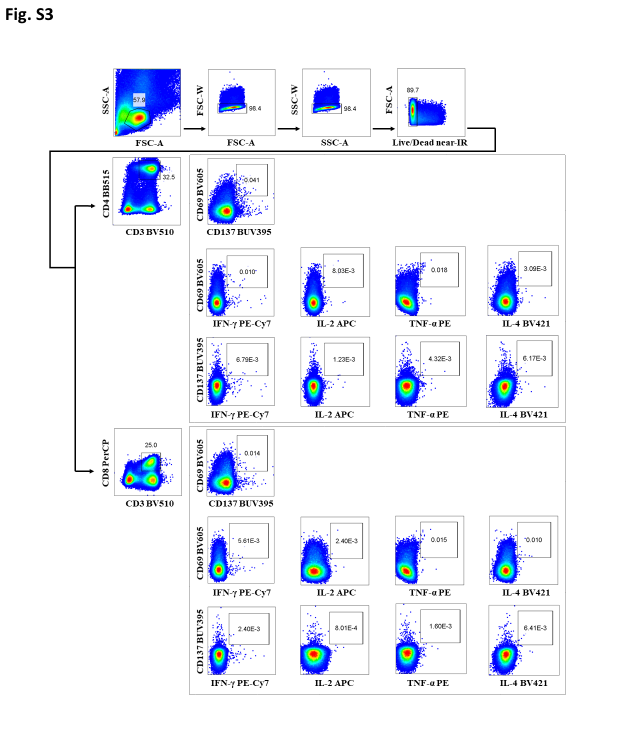


**Supplementary Figure 1.** *Lee CM et al.*


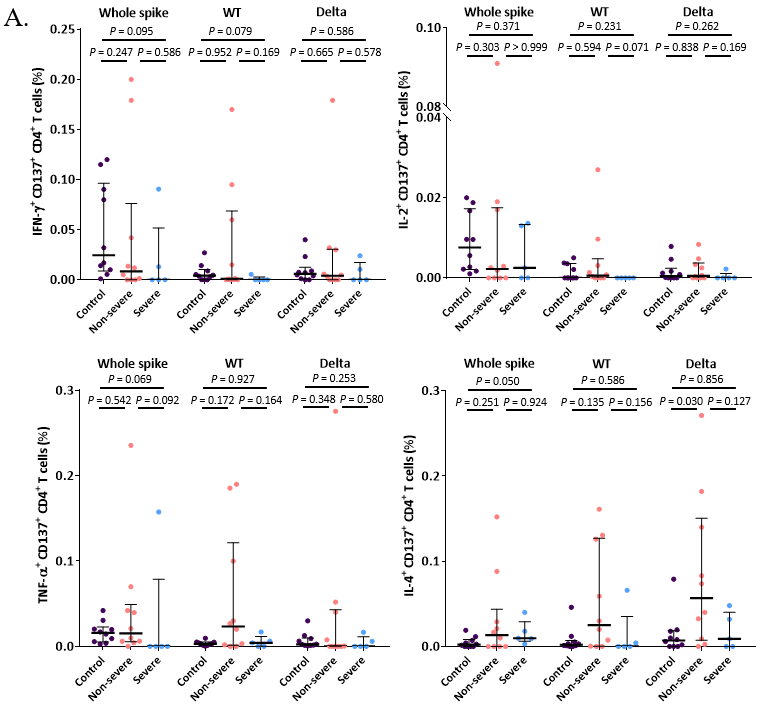


**Supplementary Figure 2A.** *Lee CM et al.*


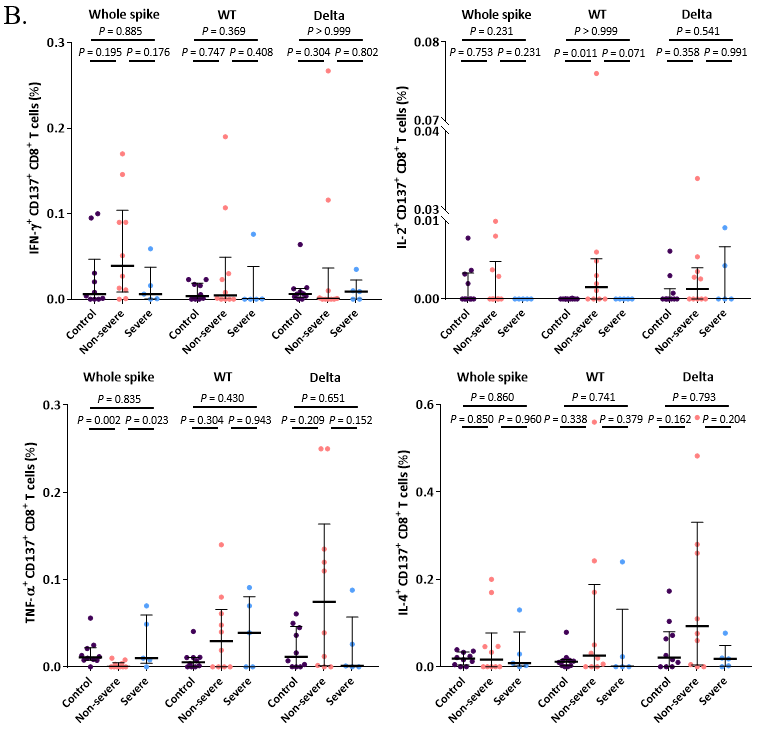


**Supplementary Figure 2B**. *Lee CM et al.*
